# High-Density Balsamic Vinegar: Application of Stable Isotope Ratio Analysis to Determine Watering Down

**DOI:** 10.1021/acs.jafc.2c08362

**Published:** 2023-03-07

**Authors:** Matteo Perini, Silvia Pianezze, Mauro Paolini, Roberto Larcher

**Affiliations:** † Fondazione Edmund Mach, Via E. Mach 2, San Michele all’Adige, 38098 Trento, Italy

**Keywords:** balsamic vinegar of Modena (ABM), stable isotope ratio analysis, watering down, δ^18^O

## Abstract

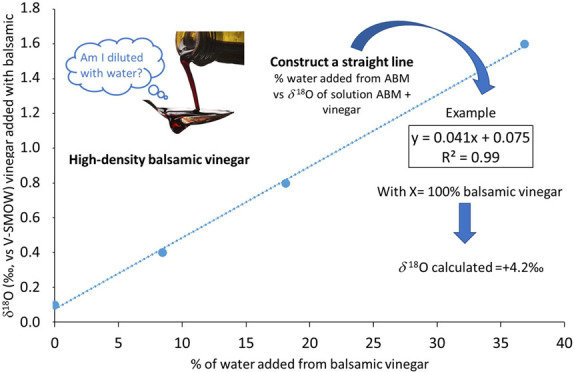

Balsamic vinegar of Modena (ABM) is a product obtained from concentrated grape must with the addition of wine vinegar. It can be adulterated with the addition of exogenous water. The official method EN16466-3, based on the analysis of the stable isotope ratio *δ*^18^O of the water, is not applicable to ABM with high density (above 1.20 at 20 °C). In this work, for the first time, the official method was modified, providing for a prior dilution of the sample and applying a correction of the data in order to eliminate the isotopic contribution of the diluent, whereupon the within- and between-day standard deviations of repeatability (Sr) were estimated. Considering the limit values of *δ*^18^O for vinegar and concentrated must, the threshold limit of *δ*^18^O, below which the ABM product can be considered adulterated, has been identified.

## 1 Introduction

*Aceto balsamico di Modena IGP* (ABM) is an Italian PGI (Protected Geographical Indication) vinegar appreciated worldwide, which is obtained from cooked and/or concentrated grape must (at least 20% of the volume) with the addition of at least 10% of wine vinegar and a maximum 2% of caramel for color stability.^1^ Although the Italian origin of the raw material is not prescribed in the production specification,^1^ the grapes of the partially fermented and/or cooked and/or concentrated grape must should come from the following typical Italian grapevine varieties: Lambrusco, Sangiovese, Trebbiani, Albana, Ancellotta, Fortana, and Montuni. On the contrary, the vinegar can be of national or foreign origin.

The balsamic vinegar market was valued at USD 2.32 billion in 2021 and is expected to reach USD 2.96 billion by 2029. Consumers are willing to pay a lot for a bottle of authentic Italian balsamic vinegar, especially the high-density one that most closely resembles Traditional Balsamic Vinegar of Modena (a Protected Origin Designation product). This high demand has created a profitable market for companies from all sectors of the food industry^2^ and has exposed this high value-added product to counterfeiting and imitation by unscrupulous Italian and foreign producers (also by exploiting the so-called “Italian sounding”).^3^ Among adulterations, the use of vinegar obtained from the fermentation of sugars other than those of grapes (such as cane and/or beet) or obtained from the acetic fermentation of diluted “raisin wine” is the most frequent occurrence. This “raisin vinegar”, commonly produced in some Mediterranean countries by fermenting dried grapes and rehydrating with tap water, is improperly imported into Italy as “wine vinegar” specified by the Directorate General of Agriculture and Rural Development of the European Commission and by the European Commission (note No. 3284; written questions E-1690/02 and E-1506/02). In fact, according to European Regulations wine vinegar is a product obtained only from the acetous fermentation of wine, which is in turn defined as the product obtained exclusively from the alcoholic fermentation of fresh grapes, whether crushed or not, or grape must (EC 479/2008 e annex IV points 1 and 17). In addition to the fraudulent use of this raisin vinegar, adulterations of ABMs can also occur with water. Both ABM’s starting ingredients (must and vinegar) may have been watered down before mixing.

Since 2013, the European Committee for Standardization (CEN) has issued a method for determining the water fraudulently added to the vinegar (EN16466-3 ^18^O-IRMS). The method is based on the stable isotope ratio analysis of the bulk vinegar (expressed as *δ*^18^O in ‰ with regard to the international standard V-SMOW2). As it was not specified in the official method, Camin et al. proposed a methodological study in which the *δ*^18^O threshold limit for wine vinegar was established. Threshold *δ*^18^O values of −2‰ and −5‰ have been fixed for wine vinegar having an acetic acid content higher and lower than 9%, respectively.^4^ Perini et al. have demonstrated the applicability of the CEN method even to the more complex balsamic vinegar matrix without significant variations in terms of repeatability.^5^ Moreover, the organization of a specific intercollaborative study, with the participation of seven different laboratories, allowed the definition of validation parameters for the oxygen stable isotopic ratios of balsamic vinegar water.^6^

The density of the ABM must be greater than 1.06,^1^ but balsamic vinegars with very high density (greater than 1.20) are commercially available. They are obtained by adding a high amount of concentrated must, whose density cannot be less than 1.24 at a temperature of 20 °C, or thanks to a long product aging in the barrel, which leads to intense evaporation and concentration. Products with such high density cannot be analyzed by using the official method reported in the EN16466-3 ^18^O-IRMS. Indeed, the high density of the product affects the base principle of the analysis, that is, the equilibration between CO_2_ and the water in the sample.

In this work, the official method has been implemented and the within- and between-day standard deviations of repeatability (Sr) have been calculated. The samples were diluted prior to the analysis and a data correction to eliminate the diluent isotopic contribution was applied. Considering the limit value of *δ*^18^O for a nonwatered product reported in the literature for vinegar and for rectified concentrated must,^4,7^ the threshold limit of *δ*^18^O, below which the ABM product can be considered as adulterated, was identified.

## 2 Materials and Methods

### 2.1 Samples and Moisture Analysis

A wine vinegar sample having an acidity of 10.5% was mixed with increasing quantities of high-density balsamic vinegar. Vinegar was chosen as the diluent, since it is the basic matrix of the balsamic vinegar and is easier to mix than water. In fact, in the tests carried out, the latter tends to form a nonhomogeneous solution with the high-density balsamic vinegar whose sugar component, due to the high concentration, tends to caramelize.

Two tests were carried out using two different ABMs with different density (sample A = 1.29 and sample B = 1.26). The relative humidity (RH) of vinegar and balsamic vinegar samples was measured using the method reported by Bradley and Vanderwarn.^8^

In order to calculate the within-day repeatability of the method, the test, as described in the following point 3.2, was repeated ten times for the same sample of vinegar-ABM solution. To evaluate the between-day (or extended) repeatability of the method, the same experiment (see 3.2) was also carried out on the same vinegar-AMB solution on three different days, one month apart.

In a second experiment, two different fresh grape musts were concentrated, up to a density = 1.30, under high-vacuum evaporation as reported by Guyon et al.^9^ Wine vinegar sample having an acidity of 10.5% was mixed with increasing quantities of the prepared concentrated grape musts (CMs) after measuring their humidity.

### 2.2 *δ*^18^O Stable Isotope Analysis

The ^18^O/^16^O ratio analyses of grape vinegar and balsamic vinegar and their mix were performed using an Isotope Ratio Mass Spectrometer (IRMS) (SIRA II, VG Fisons, Middlewich, U.K.) connected to a water/CO_2_ equilibration system (Isoprep 18, VG Fisons). The analytical setup is described in the EN16466-3 ^18^O-IRMS method for grape vinegar.

According to the IUPAC protocol,^10^ the ^18^O/^16^O values are expressed in the delta scale (δ‰), against the international standards V-SMOW2/SLAP (Vienna-Standard Mean Ocean Water/Standard Light Antarctic Precipitation-International Atomic Energy Agency, Vienna, Austria) for oxygen as per eq 1:
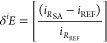
1where *i* is the mass number of the heavier isotope of element E (for example, ^18^O); *R*_SA_ is the respective isotope ratio of a sample (for example, for O: number of ^18^O atoms/number of ^16^O atoms or as approximation ^18^O/^16^O); and *R*_REF_ is the respective isotope ratio of internationally recognized reference material. The delta values were multiplied by 1000 and expressed in units “per mil” (‰). Each sample was analyzed in duplicate.

The isotopic values were calculated against a working in-house standard water that was itself calibrated against international reference materials V-SMOW2 (0‰ ± 0.02) and V-SLAP (−55.5‰ ± 0.02) (IAEA, Vienna, Austria).

## 3 Results and Discussion

### 3.1 Analysis of *δ*^18^O in High Density Balsamic Vinegar (ABM)

Table 1 shows the calculations related to the analysis carried out in two separate tests (samples A and B). In every test, the same wine vinegar, used as a diluent, was mixed with increasing amounts of one of the two high-density balsamic vinegars. The amount of water (expressed in g) added to the wine vinegar by mixing it with the balsamic vinegar was calculated. For the calculation, the amounts of wine vinegar and ABM used to obtain the solution and the humidity data measured for both ingredients were considered (RH = 47.6% and RH = 52.9% for sample A and B, respectively).

**Table 1:** Example of Calculation of the Percentage of Water Added to a Wine Vinegar–AMB Solution Deriving from the Addition of ABM^a^

	wine vinegar (g)	water (vinegar) humidity 89.5% (g)	balsamic (g)	water (balsamic) added humidity 47.6% (g)	water (total) (g)	percentage of water added (from balsamic)
sample A	6.03	5.40	1.05	0.50	5.90	**8.47**
	4.95	4.43	2.06	0.98	5.41	**18.11**
	3.34	2.98	3.66	1.74	4.73	**36.78**

	wine vinegar (g)	water (vinegar) humidity 89.5% (g)	balsamic (g)	water (balsamic) added humidity 52.9% (g)	water (total) (g)	percentage of water added (from balsamic)
sample B	5.98	5.35	1.05	0.56	5.91	**9.47**
	4.90	4.39	2.11	1.11	5.50	**20.18**
	3.53	3.16	3.52	1.86	5.02	**37.05**

a Two different experiments (sample A and sample B) are reported.

**Figure 1:**
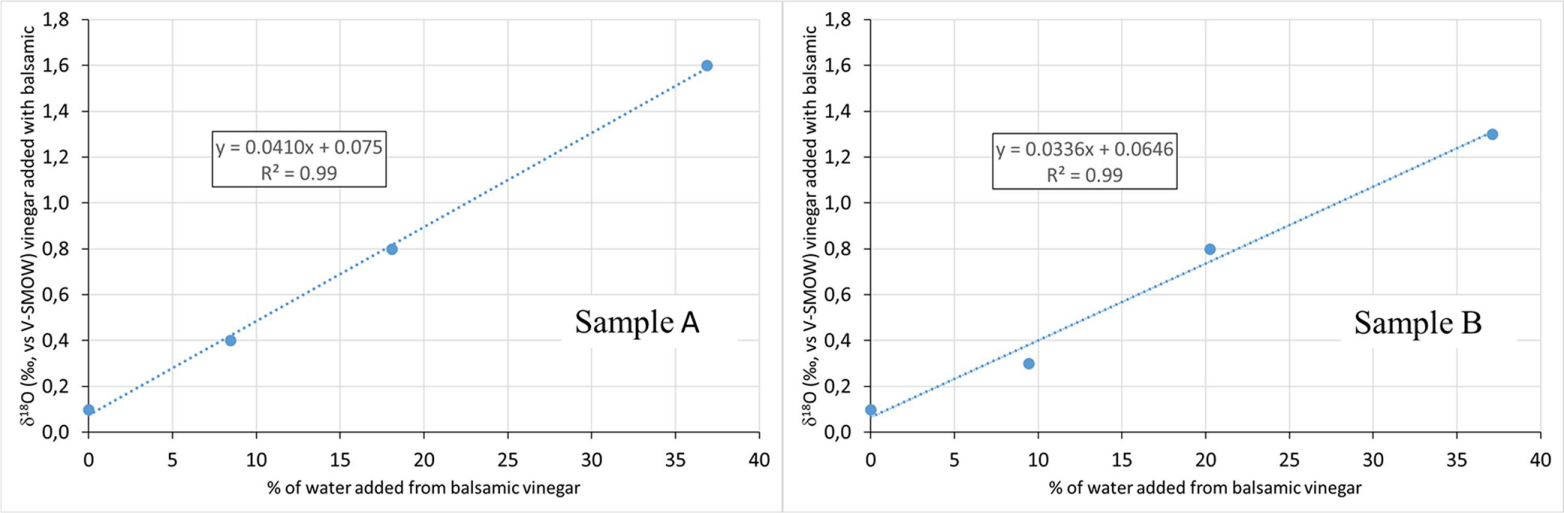
Example of correlation between % of water from balsamic vinegar added to the wine vinegar–AMB solution and *δ*^18^O of the solution. Two different example tests are reported.

The *δ*^18^O isotope ratios of the four solutions were measured and the values were plotted in Figure 1 in relation to the calculated percentages of added water (deriving from the addition of ABM to wine vinegar) ranging from 0 to about 37%. The straight trendlines obtained have an R^2^ = 0.99. By applying a linear interpolation, through the use of the equations reported in Figure 1 (for samples A and B), it is possible to calculate the expected *δ*^18^O value for a 100% solution of the two high density ABMs. In the experiments reported, for the ABM sample A the calculated value is equal to +4.2‰, while for sample B it is equal to +3.4‰.

### 3.2 Analysis of *δ*^18^O in Concentrated Grape Must (CM)

**Figure 2:**
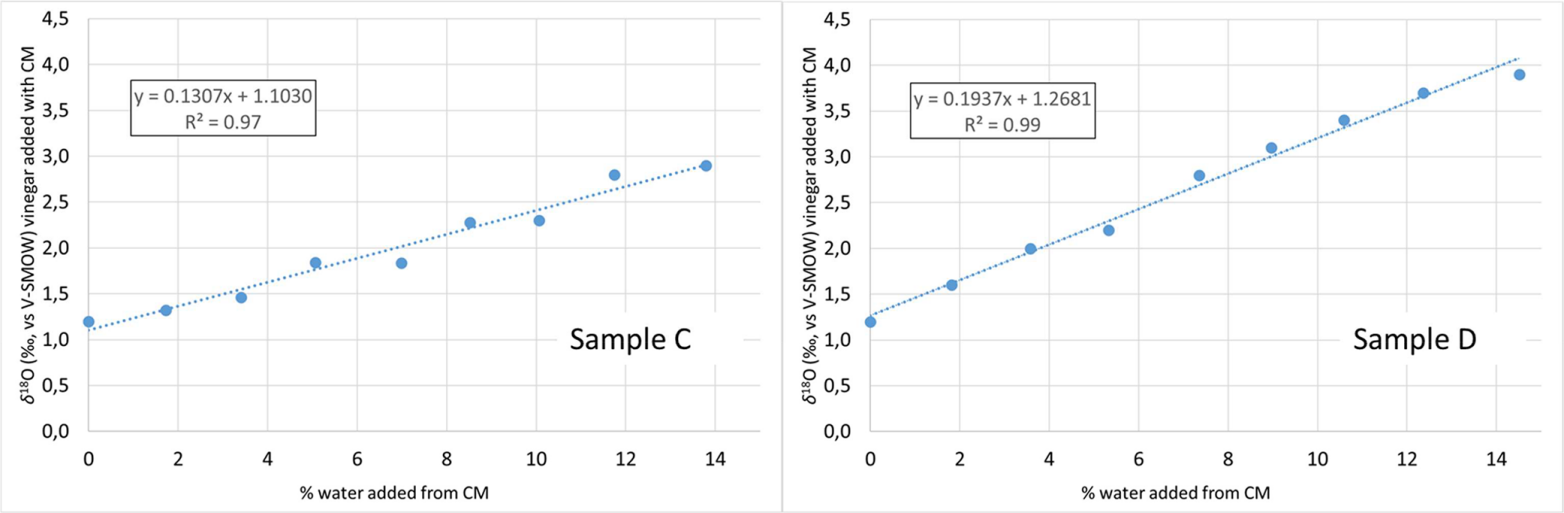
Example of correlation between % of water from concentrated grape must added to the wine vinegar–CM solution and *δ*^18^O of the solution. Two different example tests are reported.

In a second experiment, two fresh grape musts with a *δ*^18^O respectively of −1.3‰ (sample C) and +2‰ (sample D) were concentrated to obtain two concentrated grape musts (CMs) with a density of 1.30. Eight different solutions, having percentages of added water (deriving from the addition of the prepared CM to wine vinegar) ranging from 0 to about 14%, were set up. Percentages were calculated in the same way as section 3.1, considering RH = 34.4% and RH = 36.2% for samples C and D, respectively. The *δ*^18^O isotope ratio of the solutions was measured and the values plotted in Figure 2 in relation to the concentrations of added water. A *δ*^18^O value of +14.2‰ (sample C) and +20.6% (sample D) were calculated by applying linear interpolation through use of the equations shown in Figure 2. This high value of *δ*^18^O, compared to the starting *δ*^18^O of fresh must, is compatible with the kinetic fractionation due to the evapotranspiration effect of the water, which occurs during the concentration of the fresh must.^9^

### 3.3 Within- and between-Day Repeatability Estimation

The same high-density ABM sample was analyzed ten times by applying the described method (see section 3.1). To calculate each of the *δ*^18^O values reported in Table 2, four solutions were prepared each time with increasing concentrations of ABM added to the wine vinegar (from 0 to 40%), and the relative interpolation line was constructed.

**Table 2:** Within- and between-Day Repeatability of the Method

	*δ*^18^O (‰, vs V-SMOW)	date	*δ*^18^O (‰, vs V-SMOW)
1	1.5	2 October	1.5
2	1.6	17 October	1.6
3	1.5	31 October	1.6
4	1.5		
5	1.8		
6	1.6		
7	1.7		
8	1.5		
9	1.6		
10	1.7		
mean	1.6	mean	1.6
within-day Sr	0.1	between-day Sr	0.1

On the basis of the results thus obtained (Table 2), it is possible to estimate a within-day repeatability standard deviation (within-day Sr) of 0.1‰. Preparation of the vinegar–ABM solutions of the same ABM sample and use of the same wine vinegar as diluent was carried out as described above on three different days in a month. On the basis of the results obtained, it is possible to estimate a between-day (or extended) standard deviation of repeatability (between-day Sr) of 0.1‰. As prescribed by the standard ISO 21748:2017, in the absence of reproducibility that requires an intercollaborative study between laboratories, the extended repeatability herein reported can still be useful for estimating the uncertainty of this method.

### 3.4 Threshold *δ*^18^O Limit for an Authentic Balsamic Vinegar

As prescribed in Regulation (EC) No. 583/2009,^1^ balsamic vinegar is obtained from partially fermented and/or cooked and/or concentrated grape musts with a density of no less than 1.24, with the addition of a percentage of vinegar obtained by acetification of wine in the minimum measure of 10%. Furthermore, the percentage of cooked and/or concentrated grape must has to be higher than 20% of the total mass. The regulation does not lay down the minimum vinegar acidity that can be used in the formulation of the ABM. It is therefore possible to use vinegars with acidity lower than 9° (for example, diluted to 6°), whose *δ*^18^O limit value is equal to −5‰, as reported by Camin et al. for wine vinegar.^4^

As reported by Dordevic et al., the limit value of *δ*^18^O for a wine obtained from the fermentation of grapes is equal to −1.3‰.^7^ The concentrated must (CM) is normally obtained either by reverse osmosis or by high-vacuum evaporation of a fresh must. As demonstrated by Guyon et al., while the former technique has no significant fractionation effect on the oxygen isotope ratio, the latter, as also demonstrated in section 3.2, leads to an isotopic enrichment due to the evaporation of water.^9^ Unfortunately, it is not possible to know the technique by which the must was concentrated. It is therefore necessary to use the limit value reported by Dodevic et al.,^7^ assuming the use of a CM from osmosis for the formulation of the ABM.

Considering these limits and assuming an ABM formulated with 10% wine vinegar and 90% CM, it is possible to estimate a threshold value of −1.7‰. Instead, considering an ABM formulated by adding the minimum limit of 20% must with 80% wine vinegar, the threshold value drops to −4.3‰. ABM samples with δ^18^O values below this limit indicate an adulteration of the product due to the addition of exogenous water.

## Abbreviations Used

CM: concentrated grape must
ABM: Balsamic vinegar of Modena PGI
Sr: repeatability standard deviation
CEN: European Committee for Standardization
IRMS: Isotope Ratio Mass Spectrometer
